# Study on In Vitro Metabolism and In Vivo Pharmacokinetics of Beauvericin

**DOI:** 10.3390/toxins14070477

**Published:** 2022-07-12

**Authors:** Yu Yuan, Guangpeng Meng, Yuanbo Li, Chunjie Wu

**Affiliations:** 1 School of Pharmacy, Chengdu University of Traditional Chinese Medicine, Chengdu 611137, China; crystalyy520@163.com; 2 Chengdu Sintanovo Biotechnology Co., Ltd., Chengdu 611000, China; mengguangpeng@sintanovo.com (G.M.); liyuanbo@sintanovo.com (Y.L.)

**Keywords:** beauvericin, metabolism, liver microsomes, pharmacokinetic profiles

## Abstract

Beauvericin (BEA) is a well-known mycotoxin produced by many fungi, including *Beaveria bassiana*. The purpose of this study was to evaluate the in vitro distribution and metabolism characteristics as well as the in vivo pharmacokinetic (PK) profile of BEA. The in vitro metabolism studies of BEA were performed using rat, dog, mouse, monkey and human liver microsomes, cryopreserved hepatocytes and plasma under conditions of linear kinetics to estimate the respective elimination rates. Additionally, LC-UV-MS*^n^* (*n* = 1~2) was used to identify metabolites in human, rat, mouse, dog and monkey liver microsomes. Furthermore, cytochrome P450 (CYP) reaction phenotyping was carried out. Finally, the absolute bioavailability of BEA was evaluated by intravenous and oral administration in rats. BEA was metabolically stable in the liver microsomes and hepatocytes of humans and rats; however, it was a strong inhibitor of midazolam 1′-hydroxylase (CYP3A4) and mephenytoin 4′-hydroxylase (CYP2C19) activities in human liver microsomes. The protein binding fraction values of BEA were >90% and the half-life (T_1/2_) values of BEA were approximately 5 h in the plasma of the five species. The absolute bioavailability was calculated to be 29.5%. Altogether, these data indicate that BEA has great potential for further development as a drug candidate. Metabolic studies of different species can provide important reference values for further safety evaluation.

## 1. Introduction

Beauvericin (BEA) is an emerging and globally abundant mycotoxin that is produced as a secondary metabolite by several toxigenic fungi, including *Fusarium* spp., *Beauveria bassiana*, and some *Isaria* spp. [1,2]. As a mycotoxin, it is a natural contaminant of food and feed commodities [3]. Importantly, it has attracted extensive attention due to its biological activities, including anticancer, antimicrobial, insecticidal, nematocidal, phytotoxic, and antiviral activities [4]. It has been reported that BEA exerts its anticancer effects by inducing apoptosis in several cancer cell lines, including CCRF-CEM leukemia cells, human non-small cell lung cancer A549 cells, human colon adenocarcinoma Caco-2 cells, and H4IIE hepatoma cells [5,6,7,8]. BEA also displays antibacterial activities against many microorganisms, including Gram-positive and Gram-negative bacteria [9]. Moreover, BEA can simultaneously target drug resistance and morphogenesis, thus providing a promising strategy to combat life-threatening fungal infections [10]. In addition, BEA has recently been found to attenuate melanogenesis by regulating both the cAMP/PKA/CREB and LXR-α/p38 MAPK signaling pathways, consequently leading to a reduction in melanin levels [11].

**Figure 1:**
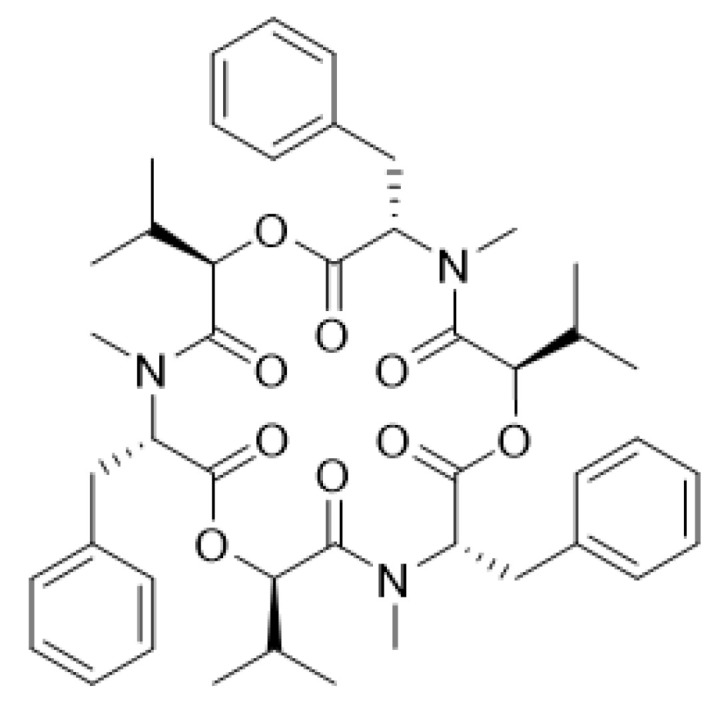
The chemical structure of BEA.

BEA is a cyclic hexadepsipeptide that belongs to the enniatin antibiotic family. Its molecular structure consists of alternating D-hydroxyisovaleryl and *N*-methyl-phenylalanyl residues (Figure 1) [1]. BEA is characterized by the existence of free electron pairs (in the oxygen carbonyl group and the tertiary amino group), which can act as a nucleophile to form ion–dipole interactions with electrophilic compounds. Because of these characteristics, BEA is an ionophore that can regulate the translocation of specific ions through cellular channels. It also can increase the intracellular Ca^2+^ concentration, which results in a difference between the intracellular and extracellular concentrations, thus leading to cell death [12,13,14,15,16]. Therefore, it was speculated that the mechanism of action of BEA is via the destruction of the cell membrane, but this mechanism remains inconclusive. Nevertheless, due to its unique structure and valuable biological functions, the cyclic hexadepsipeptide BEA has emerged as a promising agent in the pharmaceutical field [17].

Furthermore, it cannot be ignored that absorption, distribution, metabolism, and excretion (ADME) data, particularly on emerging mycotoxins, are still scarce. Hence, there is an urgent need for a deeper comprehension of the potential medicinal properties of BEA [18,19]. In recent years, there have been some reports on the ADME data of BEA. For example, studies by Li and others have shown that BEA is a strong inhibitor of midazolam 1′-hydroxylase (CYP3A4/5) and mephenytoin 4′-hydroxylase (CYP2C19) activities in human liver microsomes (HLMs) as well as CYP3A1/2 in rat liver microsomes (RLMs). In addition, with an increasing dose, the exposure of BEA in rats has been found to be enhanced [20]. Other drug studies have demonstrated that BEA is present not only in several organs and tissues but also in the serum, confirming drug absorption through the visceral peritoneum into the portal vein after intraperitoneal administration. Therefore, it has been suggested that BEA may be useful as a novel therapeutic agent for cancer treatment because it was only detected in the tumor tissues [21].

It is often challenging for cyclic hexadepsipeptide compounds such as BEA to become successful drugs due to multiple ADME issues such as limited metabolic stability, poor oral bioavailability, and a short half-live (T_1/2_) [14]. To meet these challenges, early preclinical in vitro and in vivo studies such as metabolic stability in liver microsomes, hepatocytes, and plasma as well as protein binding and bioavailability assays are highly desirable. On the other hand, in vitro metabolic profiling data derived from animal species can strengthen the prediction of the metabolic profile in humans [21]. The difference in the in vitro metabolic profiles in various species in preclinical studies is closely related to the exposure and safety of a drug in humans [22]. However, the pharmacokinetic (PK) properties and metabolic stability of BEA in different species have not yet been characterized.

The aim of the present study was to summarize the in vitro distribution and metabolism (DM) and in vivo PK data of BEA in different species in order to predict human pharmacokinetics and support BEA’s advancement into clinical trials. Herein, the main DM properties of BEA in humans, rats, mice, dogs, and monkeys were determined by estimating its binding rate to plasma proteins; the inhibitory potentials of BEA against drug-metabolizing enzymes such as cytochrome P450 (CYP); the metabolic stability of BEA in plasma, liver microsomes, and hepatocytes; and its metabolite identification. In addition, the resulting PK properties in rats were analyzed.

## 2. Results

### 2.1. In Vitro Plasma Protein Binding (PPB)

As listed in Table 1 and Table S2, the in vitro results of PPB of the reference compound warfarin were 98.86 ± 4.99%, 99.48 ± 5.60%, 97.29 ± 6.32%, 97.54 ± 7.2%, and 99.16 ± 9.69% in human, rat, mouse, dog, and monkey plasma, respectively; and these findings are consistent with historical data [22]. The recovery of BEA in the five species was greater than 70%. BEA showed high binding to human, rat, mouse, dog, and monkey plasma proteins, with bound fractions of 99.88 ± 3.53%, 99.93 ± 2.90%, 99.94 ± 0.57%, 99.92 ± 2.91%, and 99.91 ± 1.62%, respectively. These results suggest that BEA has a high PPB affinity.

### 2.2. CYP Inhibition in HLMs

**Figure 2:**
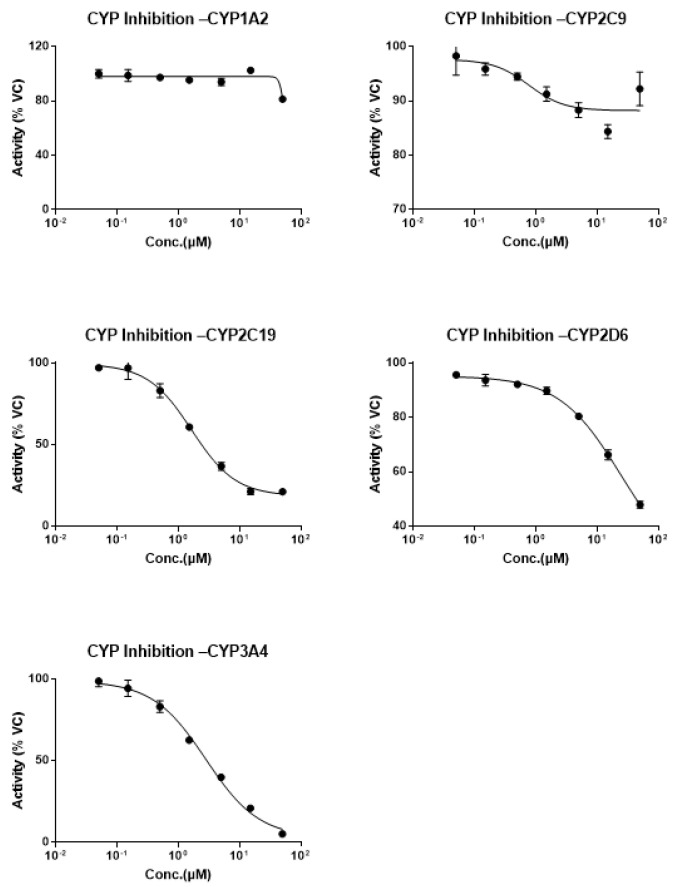
Line graph of CYP inhibition evaluation of BEA.

In this study, the inhibitory potential of selective inhibitors of BEA depletion in HLMs was determined. The CYP inhibition results of BEA are presented in Table 2 and Figure 2. The half-maximal inhibitory concentration (IC_50_) values of BEA for the inhibition of phenacetin (CYP1A2), diclofenac (CYP2C9), and dextromethorphan (CYP2D6) were >10 μM in HLMs, suggesting that BEA is a weak inhibitor of these three CYP enzymes. However, BEA showed strong inhibition of CYP3A4 (midazolam) and CYP2C19 (s-mephenytoin), with IC_50_ values of 2.24 μM and 3.30 μM, respectively. These findings indicate that BEA is a potent inhibitor of CYP3A4 and CYP2C19. In addition, the IC_50_ values for the standard positive control inhibitors were consistent with the in-house historical values (Table 2 and Table S3).

### 2.3. Metabolic Stability in Liver Microsomes

**Figure 3:**
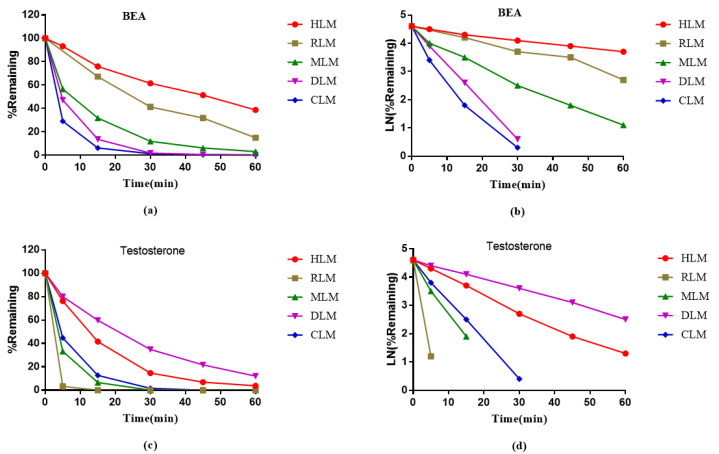
The metabolic stability curve (**a**,**c**) and the regression equation of the linear part of the curve (**b**,**d**) of BEA and Testosterone in liver microsomes of human, rat, mouse, dog and monkey.

Metabolic stability assays of BEA were performed in liver microsomes of human, rat, mouse, dog, and cynomolgus monkey (HLMs, RLMs, MLMs, DLMs, and CLMs, respectively) at a final concentration of 1 μM. The metabolic stability results in liver microsomes of the five species are presented in Table 3 and Figure 3. The remaining amounts of BEA at 60 min were 38.8%, 14.9%, 3.1%, 0.3%, and 0.3% in HLMs, RLMs, MLMs, DLMs, and CLMs, respectively. Although the T_1/2_ values of BEA were shorter in MLMs, DLMs and CLMs, BEA was stable in HLMs with T_1/2_ > 30 min and CL_int(liver)_ < 30 mL/min/kg. The remaining amounts of the positive control (testosterone) at 60 min were 3.8%, 0%, 0%, 12.0% and 0% in HLMs, RLMs, MLMs, DLMs, and CLMs, respectively, which is consistent with historical data.

### 2.4. Metabolic Stability in Hepatocytes

**Figure 4:**
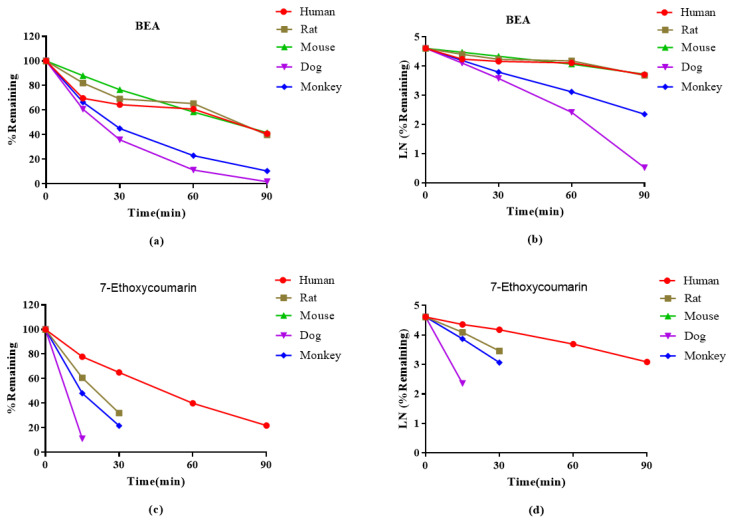
The metabolic stability curve (**a**,**c**) and the regression equation of the linear part of the curve (**b**,**d**) of BEA and 7-Ethoxycoumarin in human, rat, mouse, dog and monkey hepatocytes.

The metabolic stability of BEA in hepatocytes of humans, rats, monkeys, dogs, and mice was studied. The results are presented in Table 4 and Figure 4. After incubation for 90 min, the remaining fractions of BEA in hepatocytes of humans, rats, mice, dogs, and monkeys were 40.9%, 39.8%, 41.7%, 1.7%, and 10.5%, respectively. BEA was stable in human, rat, and mouse hepatocytes (T_1/2_ > 60 min), and the CLint (liver) (well-stirred model) were 46.3, 85.7, and 229.7 mL/min/kg, respectively. The in vitro results of the control compounds show high clearance, which is consistent with historical data.

### 2.5. Plasma Stability

**Figure 5:**
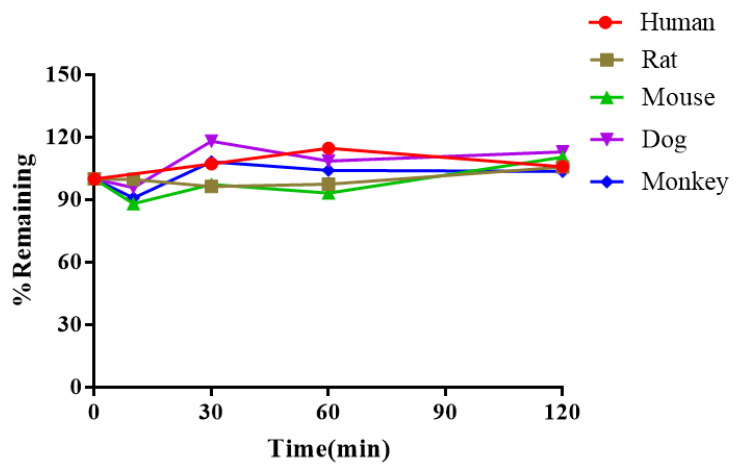
Stability of BEA in the plasma of five species.

Plasma stability plays an important role in drug discovery and development. Unstable compounds tend to have a rapid clearance and a short T_1/2_, resulting in a poor in vivo performance. The metabolic stability of BEA in human, rat, mouse, dog, and monkey plasma is shown in Table 5 and Figure 5. The remaining concentrations of BEA were found to be 106.0%, 105.8%, 110.6%, 113.1%, and 103.7% after incubation for 120 min in human, rat, mouse, dog and monkey plasma, respectively. The T_1/2_ of BEA in the plasma of the five species was approximately 5 h. The control compounds all showed a short T_1/2_ in the plasma of the five species (Table S4 and Figure S1).

### 2.6. Metabolite Identification

**Figure 6:**
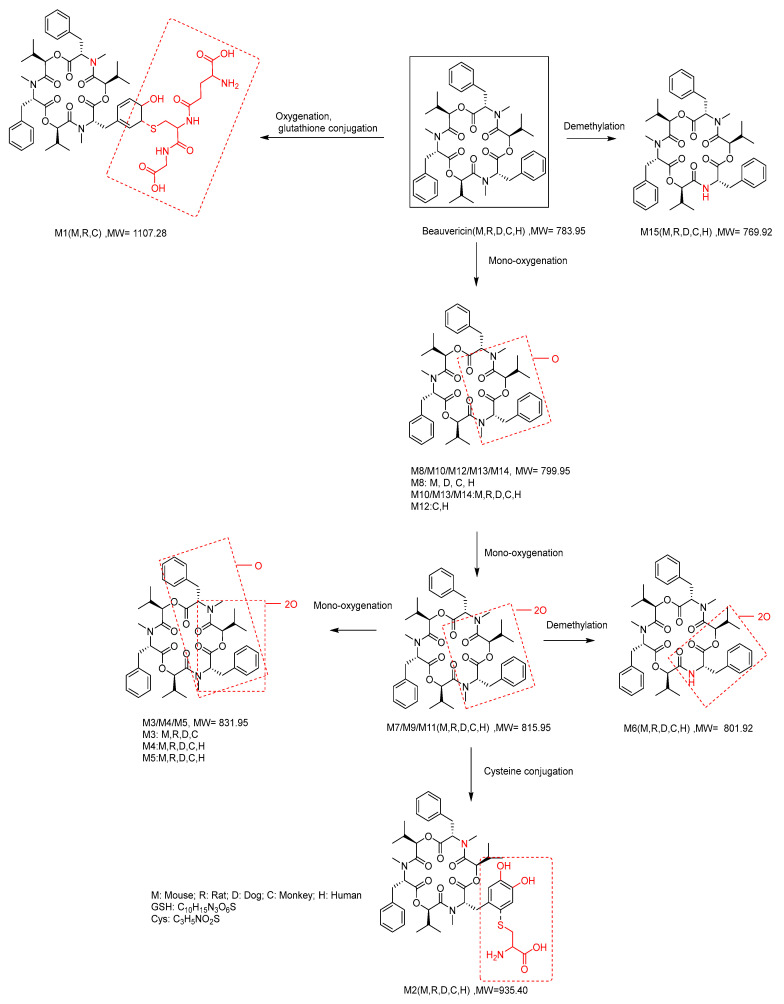
Metabolic pathways of BEA in mouse, rat, dog, monkey and human liver microsomes (M, R, D, C and H mean mouse, rat, dog, monkey, human, respectively).

In addition to the unchanged BEA (MW = 783.95), a total of 15 metabolites of BEA were detected and identified by LC-UV-MS *^n^*(*n* = 1~2) from mouse, rat, dog, monkey and human liver microsomes (Table 6). The metabolites were assigned as M1: oxygenation and glutathione conjugation metabolite (MW = 1107.27, P + O + GSH); M2: di-oxygenation, cysteine conjugation metabolite (MW = 935.09, P + 2O + Cys); M3/M4/M5: tri-oxygenation metabolites (MW = 831.95, P + 3O); M6: di-oxygenation, *N*-demethylation metabolite (MW = 801.92, P + 2O − CH_2_); M7/M9/M11: di-oxygenation metabolites (MW = 815.95, P + 2O); M8/M10/M12/M13/M14: mono-oxygenation metabolites (MW = 799.95, P + O); and M15: *N*-demethylation metabolite (MW = 769.92, P − CH_2_). Fifteen metabolites (M1~M15) were identified in CLM, while only 13 metabolites were detected in MLM, RLM, HLM. The proposed metabolic pathways of BEA in mouse, rat, dog, monkey and human liver microsomes were *N*-demethylation and mono-oxygenation (Figure 6).

### 2.7. In Vivo PK Studies

**Figure 7:**
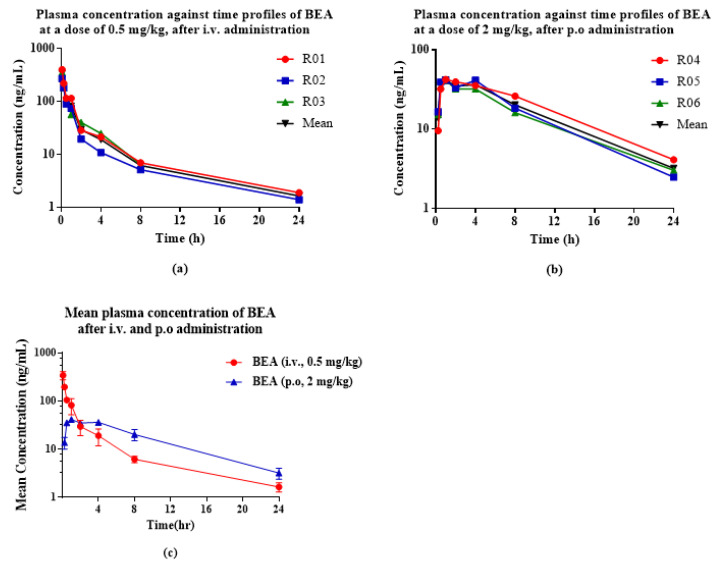
Plasma concentration–time profiles of BEA at 0.5 mg/kg after i.v. administration (**a**), at 2 mg/kg after p.o. (**b**) to rats. Data are represented in *n* = 3 with the mean ± SD (**c**).

Subsequently, BEA was assessed for its druglike properties, including the in vivo PK properties after a single intravenous or oral administration. The PK behavior of BEA was evaluated in Sprague Dawley rats (Table 7 and Figure 7). The liquid chromatography–tandem mass spectrometry (LC/MS/MS) method (Table S5) showed satisfactory results for the determination of BEA in rat plasma and was used for the PK study. Inspection of the plasma concentration−time profile for BEA revealed that the mean values of the maximum plasma concentration (C_max_) after intravenous(i.v.) and oral dosing(p.o.) were 454.0 ± 110.0 ng/mL and 41.6 ± 0.6 ng/mL, respectively. The areas under the plasma concentration−time curve extrapolated to the last time point (AUC_0−t_) after intravenous and oral dosing were 339.0 ± 66.8 ng·h/mL and 393.0 ± 54.6 ng·h/mL, respectively. The elimination kinetics of BEA demonstrated durable plasma T_1/2_ values of 5.1 ± 2.6 h and 5.9 ± 0.6 h, respectively. Furthermore, BEA showed a moderate rate of clearance of 23.9 ± 4.3 mL/min/kg after intravenous injection. The time to C_max_ (T_max_) was 1.0 h, indicating that BEA was absorbed rapidly after oral dosing. The absolute oral bioavailability was approximately 29.5% in rats. Together, the favorable PK properties, moderate clearance, relatively extended T_1/2_, and effective exposure level of BEA in vivo suggest that BEA is suitable for further study as a drug candidate.

## 3. Discussion

Drug metabolism is considered to be one of the most important factors affecting drug action [22]. The metabolism and inhibition of BEA in HLM and RLM were investigated by Li and others [20]. Their results showed that BEA strongly inhibited CYP3A4 and CYP2C19 in HLMs, which is consistent with our experimental results. In the in vivo PK test, the reported literature focused on the pharmacokinetics of BEA in rats after p.o administration and co-administration with ketoconazole, which indicated that a pharmacodynamic function may play a role in the synergistic effect on antifungal activity. In addition, Li Mei’s group also studied the inhibition mechanism of CYP enzymes and determined that the mechanisms of BEA inhibiting CYP3A4 and CYP2C19 are mix-type and competitive kinetics, respectively. Considering that as a cyclic hexadepsipeptide compound BEA is often difficult to make into a successful drug due to its limited metabolic stability, poor oral bioavailability, short half-live (T_1/2_) and high toxicity [13], studies on the metabolic pathways and stability of BEA, the enzymes and kinetic parameters involved in its metabolism, drug interactions caused by its metabolism, and metabolites of BEA are essential to ensure its safety and efficacy in order for it to become an approved drug [23]. In our study, we further defined the characteristics of BEA regarding its plasma protein binding rate, metabolic stability in liver microsomes, hepatocytes and plasma, metabolites and PK behavior through in vitro and in vivo experiments [24]. In vitro metabolism comparison tests of different species are helpful for us to estimate which species are similar to human with regard to metabolic characteristics. Therefore, these in vitro and in vivo tests will provide essential information for evaluating its metabolic stability in vivo of BEA, so as to provide reference values for preclinical safety evaluation [25].

Human PPB involves the reversible association of drugs with plasma proteins such as albumin, α_1_-acid glycoprotein, and others [26]. Drugs are in equilibrium between their protein-bound and free forms [27]. Since only free drugs exhibit the intended therapeutic effect, the PPB affinity of drugs or new chemical entities becomes a crucial property [28,29,30]. It is important to determine the PPB across different species to establish the safety margins for human exposure and appropriate doses for clinical trials. The PPB for BEA were assessed in human, rat, mouse, dog, and monkey plasma using warfarin as a positive control. BEA showed high binding to human, rat, mouse, dog, and monkey plasma proteins. No significant species-specific differences in the PPB study were observed. Thus, the data of the PPB assay are significant with respect to the toxicity, pharmacology, and PK of BEA.

For a lead compound, the in vitro determination of biotransformation pathways by CYP reaction phenotyping is a central element for assessment of the inhibition and induction potentials. In guidance documents provided by drug administration authorities, CYP enzymes such as CYP1A2, CYP2C9, CYP2C19, CYP2D6, and CYP3A4 are known as the critical enzymes for metabolism [31]. A relatively small number of CYP enzymes metabolize hundreds of drugs and other foreign compounds [32,33,34]. The ability of an individual CYP enzyme to metabolize multiple substrates is responsible for a large number of drug–drug interactions associated with CYP inhibition [35]. The majority of drugs are either substrates or inhibitors of CYP enzymes [36]. Some drugs also act as CYP inducers, thereby speeding up the metabolism of co-administered drugs [37].

As a cyclic peptide compound, BEA can be incubated with biological matrices to evaluate their stability [26,38,39]. A variety of in vitro systems derived from the human liver can be used to investigate potential drug interactions, such as subcellular human liver tissue components, recombinant CYP enzymes, and human liver tissue [40]. In this study, human liver microsomes were used to investigate the inhibitory effect of BEA on CYP enzyme. In the human liver component system, the indicator substrate method is usually used to study the inhibition mechanism of drugs on CYP enzymes (such as reversible or time-dependent inhibition). For different CYPs, specific inhibitors must be selected, and the corresponding inhibitor concentration shall be selected according to the IC_50_ calculation results under different concentrations in combination with the experimental conditions [41]. Dimethyl sulfoxide (DMSO) is usually used in the CYP inhibition test because it can not only act as a solvent for compounds, but also CYPs are very sensitive to it. Since some organic solvents can inhibit or induce enzyme activity, the organic solvent with the lowest concentration (<1% (*v*/*v*), preferably <0.5%) should be used [42]. The maximum concentration of DMSO is 0.5% in the inhitition studies [43].

In the present study, BEA displayed no significant inhibitory effects on the activities catalyzed by CYP1A2, CYP2C9, and CYP2D6. However, BEA was shown to be a strong inhibitor of CYP3A4 and CYP2C19 in HLMs. These results are consistent with those of previous reports, further confirming that exposure of BEA may be influenced by CYP inhibitors, in particular by inhibitors of CYP3A4 or CYP2C19, potentially affecting the safety or efficacy of BEA [44]. Interestingly, voriconazole, an antifungal drug, is a strong inhibitor of CYP3A4 and CYP2C19, but it also acts as a substrate of these two CYP enzymes [45]. As they are considered to influence elimination, patients who take voriconazole with other substrate drugs of these two enzymes often need to have an adjusted dose and/or frequency of administration of these drugs [46]. Therefore, the CYP inhibition assay results suggest that the co-administration of other drugs that are either CYP3A4 or CYP2C19 substrates or inhibitors may affect the elimination of BEA, thus contributing to the knowledge of its metabolism. More importantly, these results also provide a reference for clinical PK research design.

Metabolic stability is commonly measured in vitro during the drug discovery process as soon as a new compound is synthesized. These data provide feedback that alerts the project team to metabolic limitations and guides metabolic stability improvement through structural modifications [23]. Reactive metabolites can affect the overall toxicity profile and have to be assessed with regard to exposure, T_1/2_, matrix of occurrence, and toxicity mechanism [47]. In addition to clarifying drug clearance, cross-species comparisons of drug metabolic profiles are also important from the drug safety standpoint.

Clearance may also be affected by extramicrosomal metabolism, renal clearance, biliary extraction, and hydrolysis in the plasma or intestine. In this study, the metabolic stability of BEA in hepatocytes and liver microsomes of different species was investigated using the substrate elimination method, and the main influencing factors of its kinetic properties were evaluated by the remaining fraction (%) and T_1/2_. The metabolic stability of BEA in liver microsomes was determined for phase I oxidative reactions. It was demonstrated that BEA was stable in HLMs, with a T_1/2_ > 30 min and CL_int(liver)_ < 30 mL/min/kg. In addition, the metabolic stability of BEA in RLMs was similar to that of HLMs, compared to those of other species. As shown in Table 3, the metabolism of BEA in dogs and monkeys in vitro showed a quick degradation process that was different from that of the other species. The metabolic stability of BEA in liver microsomes is consistent with that in hepatocytes. BEA is metabolized most rapidly in hepatocytes of monkeys, mice and dogs, and slowly in hepatocytes of humans and rats.

Further, in the metabolite identification study, 13~15 metabolites of BEA were identified from liver microsomes of the different species. The qualitative and quantitative results in the form of metabolic profiles were observed to be similar in rat and human liver microsomes. As shown in Table 3, Figure 3 and Table 6, the metabolism of BEA in dogs, mice and monkeys in vitro showed a quick degradation process, which was different from the other species. M7 (di-oxygenation) and M9 (di-oxygenation) were the main metabolites in the five species. The proposed metabolic pathways of BEA in mouse, rat, dog, monkey and human liver microsomes were *N*-demethylation and mono-oxygenation. In vitro metabolic and plasma protein binding data for animals and humans should be evaluated before initiating human clinical trials [48]. The analytical data in vitro showed that there are still some differences in the metabolic rates between rodents and humans, which provides an important basis for us to choose rats to predict drug metabolism in vivo.

In the liver microsomes system, the compounds are completely exposed to the enzyme system, while in the hepatocyte system, the compounds need to enter the cells to be metabolized. Therefore, in general, if the compounds cannot fully penetrate the membrane, some compounds cannot enter the hepatocytes to be metabolized, which is why the result of remaining hepatocytes is higher than that of liver microsomes in some species [49].

Unstable compounds often have a high clearance and a short t_1⁄2_, resulting in poor in vivo PK and a disappointing pharmacological performance. Plasma degradation clearance can be overlooked if we only focus on microsomal stability. Therefore, it is important to anticipate and assess early plasma stability. The T_1/2_ of BEA in the plasma of five species was about 5 h (Table 5).

As a cyclic peptide compound, even if BEA has highly effective and selective pharmacological activities as expected, preclinical and clinical development may fail because of its adverse physicochemical and PK properties [23]. The clearance rate of BEA in rats was equivalent to the metabolic stability of liver microsomes in vitro, and there was a correlation between the metabolic results in vivo and in vitro. The volume of distribution of BEA was 5.9 ± 2.5 L/kg (Table 7), indicating that BEA is distributed extensively to tissues [50].

Although the absolute oral bioavailability of BEA was determined to be 29.5%, it is absorbed quickly in rats and can be maintained in vivo for a long time. These PK properties are similar to those of cyclosporin, a cyclic polypeptide immunosuppressant, which was approved for clinical use in the 1980s. It is composed of 11 amino acids and produced by *B. bassiana* [51]. Therefore, to increase the oral bioavailability of BEA and to enhance its pharmacological efficacy, a lot of work should be completed to ascertain the optimal delivery system for BEA, which is important for its practical application.

## 4. Conclusions

In conclusion, BEA exhibits favorable pharmacokinetics in vitro and in vivo. The metabolic stability test of BEA in vitro indicate that BEA has a relatively stable metabolism in liver microsomes, hepatocytes of humans and rats. Importantly, the metabolic rates of rodents are similar to those of humans. The proposed metabolic pathways of BEA in mouse, rat, dog, monkey and human liver microsomes were *N*-demethylation and mono-oxygenation. BEA showed a high PPB to human, dog, monkey, rat, and mouse plasma proteins. No significant species-specific differences in the PPB study were observed. BEA demonstrated strong inhibition of CYP3A4 and CYP2C19 in HLMs, suggesting that there is a potential risk for its coadministration with other drugs. The in vitro ADME characteristics and in vivo pharmacokinetic findings on BEA are in line with a low-clearance compound which has good bioavailability after oral administration, supporting BEA’s progression to clinical investigation. Metabolic studies of different species can provide important reference values for further safety evaluation. Altogether, these results indicate that BEA has the potential for further development as a drug candidate.

## 5. Materials and Methods

### 5.1. Materials

The test compound, beauvericin (Batch Number: LL-BJJS-1906-001), with a chromatographic purity (HPLC) of more than 99%, was synthesized and characterized at Sichuan LanLi Pharmtech Co., Ltd., Cheng Du, Sichuan, China.

Dimethyl sulfoxide, hydrochloric acid, potassium chloride, disodium hydrogen phosphate, sodium dihydrogen phosphate, potassium phosphate, magnesium chloride and sodium hydroxide were procured from the Sinopharm Group (Shanghai, China). Tolbutamide, NADPH (β-nicotinamide adenine dinucleotide 2′-phosphate), α-naphthoflavone, sulfaphenazole, (±)-N-3-benzylnirvanol, quinidine, ketoconazole, and William’s E medium, testosterone and 7-ethoxycoumarin were purchased from Sigma-Aldrich (Shanghai, China). Phenacetin (CYP1A2), diclofenac (CYP2C9), S-mephenytoin (CYP2C19), dextromethorphan (CYP2D6) and midazolam (CYP3A4) were procured from Shyuanye (Shanghai, China). Fetal bovine serum (FBS) and Hank’s balanced salts of Gibco were procured from Thermo Fisher Scientific (Shanghai, China).

Mouse liver microsomes (CD-1), rat liver microsomes (Sprague Dawley), dog liver microsomes (Beagle), human liver microsomes (mixed gender) and monkey liver microsomes (Cynomolgus) were procured from either Corning or Xenotech (Shanghai, China). Cryopreserved mouse (CD-1), rat (Sprague Dawley), dog (Beagle), monkey (Cynomolgus), and human hepatocytes were procured from Bioreclamation IVT (Shanghai, China). HTDialysis^®^ 96-well Teflon equilibrium dialysis plate and cellulose membranes (12000−14000 Da molecular weight cut-off), CD-1 mouse plasma, Sprague Dawley rat plasma, beagle dog plasma, cynomolgus monkey, human plasma and warfarin were obtained from Corning (Shanghai, China).

LC-MS grade (≥99.0% pure) formic acid was purchased from J&K (Shanghai, China). HPLC-grade acetonitrile (ACN), methanol, and isopropyl alcohol were procured from Burdick & Jackson (Beijing, China). Water used for the preparation of mobile phase, rinsing solvent, and seal washes was obtained from ELGA Lab purification systems (London, UK).

### 5.2. Methods

#### 5.2.1. In Vitro Studies

##### Plasma Protein Binding

The PPB study was performed with the HTdialysis apparatus (Apricot Designs, USA). The regenerated dialysis membrane was introduced into the apparatus to create two compartments. Pooled and frozen plasma from CD-1 mice (Lot# EXR), Sprague Dawley rats (Lot# OSQ), beagle dogs (Lot# ZMB), cynomolgus monkeys (Lot# KMS), and human donors (Lot# AEL) were incubated at 37 °C for 15 min, and the pH of the plasma was adjusted to 7.4 using 0.1 M sodium phosphate and 0.15 M NaCl buffer. The plasma was spiked with BEA stock solution to obtain a final BEA concentration of 2 μM, and it was spiked with reference compound (warfarin) stock solution to obtain a final BEA concentration of 1 μM. After gentle mixing, a 100 μL aliquot of plasma was collected in acetonitrile for the T_0_ sample. A 100 μL aliquot of BEA-spiked plasma was added to one half-cell (donor compartment), and 100 μL of the blank dialysis buffer was added to the other half-cell (respective receiver compartment). The remaining BEA-spiked plasma was incubated at 37 °C and 120 rpm for 5 h in an atmosphere containing 5% CO_2_. After 5 h, the plasma and buffer samples were collected from the respective compartments and processed. The concentration of BEA was determined in all of the samples using the LC-MS/MS (6500_Triple Quad 6500 plus, AB SCIEX, Redwood City, CA, USA) method (Table S6).

The % bound was calculated as

(1) $$\%\;\mathrm{Bound}=100-100\times \frac{F}{T}$$

where

*F* = Free compound concentration, as determined by the calculated concentration on the buffer side of the membrane

*T* = Total compound concentration, as determined by the calculated concentration on the matrix side of the membrane

The percent unbound fraction (Fu) was calculated by determining the compound concentrations in the buffer and matrix compartments after dialysis according to:

(2) $$\mathrm{Fu}\;\left(\%\right)=100-\%\;\mathrm{Bound}$$

The % recovery was calculated as

(3) $$\%\;\mathrm{Recovery}=100\times \frac{\left(F+T\right)}{T0}$$

where

*T_0_* = Total compound concentration, as determined by the calculated concentration in matrix before dialysis

##### CYP Inhibition Evaluation in HLMs

The metabolic stability of BEA was evaluated against five recombinant CYP enzymes: CYP1A2 (phenacetin), 2C9 (diclofenac), 2C19 (S-mephenytoin), 2D6 (dextromethorphan), and 3A4 (midazolam). A mixture of microsomes and substrates for each CYP isozyme were prepared individually in 100 mM potassium phosphate buffer at pH 7.4. These mixtures (178 μL) were preincubated at 37 °C for 10 min, and then 2 μL of BEA stock solution prepared in a mixture of 1:1 (*v*/*v*) methyl alcohol:DMSO were added. The reaction mixture was incubated for 10 min, followed by the addition of 20 μL of 10 mM NADPH solution. The reaction plates were further incubated at 37 °C for 10 min. α-Naphthoflavone, sulfaphenazole, *N*-3-benzylnirvanol, quinidine, and ketoconazole were used as positive controls. BEA was tested at 0, 0.05, 0.15, 0.5, 1.5, 5.0, 15, and 50 μM in 0.2 mg/mL HLMs containing 10 mM NADPH. At the appropriate time point, we terminated the reaction by adding 400 µL of cold stop solution (200 ng/mL tolbutamide and labetalol in acetonitrile). The samples were centrifuged at 4000 rpm for 20 min to precipitate the protein, and then detected by LC/MS/MS. XL fit was used to plot the percent of vehicle control versus the test compound concentrations and for nonlinear regression analysis of the data. The IC_50_ values were determined using 3- or 4-parameter logistic equations. The IC_50_ values were reported as “>50 µM” when the % inhibition at the highest concentration (50 µM) was less than 50%. The results are presented in Table 2 and Figure 2.

##### Metabolic Stability in Liver Microsomes

For metabolite identification, BEA was incubated with CD-1 mouse (Lot#: 2010017), Sprague Dawley rat (Lot #: 1910100), beagle dog (Lot#: 1410114), cynomolgus monkey (Lot#: 0041001CNC), and human (Lot#: 38295) liver microsomes obtained from Corning or Xenotech. All incubations were performed at 37 °C in 100 mM potassium phosphate buffer in an orbital incubator. Various nicotinamide adenine dinucleotide phosphate (NADPH) concentrations were tried to optimize the method. Initially, NADPH (final concentration = 1 mM) (Lot#: 00616) and MLMs, RLMs, DLMs, and HLMs (protein concentration = 0.56 mg/mL) were added individually to potassium phosphate buffer and incubated for 10 min at 37 °C. Subsequently, BEA and the control were prepared with 5 μL DMSO and 495 μL acetonitrile (ACN), and then spiked into the reaction mixture to achieve a final BEA concentration of 1 μM. Samples were withdrawn from the reaction mixture at different times (5, 15, 30, 45, and 60 min), and the reaction was terminated using acetonitrile. The positive control compound testosterone was incubated in parallel with the BEA samples. Each bioanalysis plate was sealed and shaken for 10 min prior to LC-MS/MS analysis (Table S6). The equation of first-order kinetics was used to calculate T_1/2_ and CL_int(mic)_ (μL/min/mg):

(4) $$C_{t}=C_{0}\cdot e^{-k_{e}\cdot t}\;$$

when

$$C_{t}=\frac{1}{2}C_{0}\;T_{1/2}=\frac{ln2}{k_{e}}=\frac{0.693}{k_{e}}$$

(5) $${\mathrm{CL}}_{\mathrm{int}(\mathrm{mic})}=\frac{0.693}{in\;vitro\;T_{1/2}}\times \frac{1}{mg/ml\;microsomal\;protein\;in\;reaction\;systerm}$$

(6) $${\mathrm{CL}}_{\mathrm{int}(\mathrm{liver})}={\mathrm{CL}}_{\mathrm{int}(\mathrm{mic})}\times \frac{mg\;microsomes}{g\;liver}\times \frac{g\;liver}{kg\;body\;weight}$$

##### Metabolic Stability in Hepatocytes

Hepatocyte metabolic stability was evaluated in cryopreserved hepatocytes from CD-1 mice (Lot#: EXR), Sprague Dawley rats (Lot#: OSQ), beagle dogs (Lot#: ZMB), cynomolgus monkeys (Lot#: KMS), and human donors (Lot#: AEL) obtained from Bioreclamation IVT, LLC (Shanghai, China). BEA (10 mM) was incubated in duplicate in a 96-well plate containing a suspension of mouse, rat, monkey, dog, or human hepatocytes (0.5 × 10^6^ cells/mL) at 37 °C for 1.5 h in a controlled atmosphere (5% CO_2_, 95% humidified incubator). BEA and the control were prepared with 50 μL of DMSO and 450 μL of acetonitrile (ACN) to achieve a final concentration of 1 μM, and then spiked into each well of 96-well plates in duplicates. Samples were withdrawn from the reaction mixture at 0, 15, 30, 60, and 90 min and were quenched with stop solution (acetonitrile containing 200 ng/mL tolbutamide and 200 ng/mL labetalol as internal standards). A cocktail of 7-ethoxycoumarin (Lot#: I1825085) was used as the positive control for the assay. The analytical plates were sealed and stored at 4 °C until LC-MS/MS analysis. The data obtained from the BEA metabolic stability assays were used to calculate the intrinsic clearance and remaining percentages, as described in Table 4 and Figure 4.

The remaining percentages of test articles after incubation were calculated by the following equations:

(7) $$\begin{array}{l} \%Remaining\left(at\;Appointed\;Time\right)\\ =\frac{Peak\;Area\;Ratios\;of\;Test\;Article\;versus\;Internal\;Standard\;at\;Appointed\;Time}{Peak\;Area\;Ratios\;of\;Test\;Article\;versus\;Internal\;Standard\;0\;min\;} \end{array}$$

We used the equation of first order kinetics to calculate T_1/2_ and CL_int_:

Equation of first-order kinetics:

(8) $$C_{t}=C_{0}\cdot e^{-k\cdot t}\;$$

when

$$C_{t}=\frac{1}{2}C_{0},T_{1/2}=\frac{ln2}{k}=\frac{0.693}{k}$$

(9) $$\mathrm{CLint}\;\left(\mathrm{hep}\right)=\frac{k}{\mathrm{million}\;\mathrm{cells}\;\mathrm{per}\;\mathrm{mL}}$$

(10) $$\mathrm{CLint}\;\left(\mathrm{liver}\right)=\mathrm{CLint}\;\left(\mathrm{hep}\right)\times \mathrm{Liver}\;\mathrm{weight}\;\left(g/\mathrm{kg}\;\mathrm{body}\;\mathrm{weight}\right)\times \mathrm{hepatocellularity}$$

##### Plasma Stability

The pooled frozen plasma was thawed in a water bath at 37 °C prior to the experiment. Plasma was centrifuged at 4000 rpm for 5 min, and the clots, if any, were removed. Using an Apricot automation workstation, blank plasma (98 μL/well) was added to six 96-well reaction plates (Blank, T0, T10, T30, T60, and T120). An Apricot automation workstation was used to add working solution (2 μL/well, 100 μM) to all reaction plates except the Blank plate (T0, T10, T30, T60, and T120). All reaction plates containing mixtures of compound and plasma were incubated at 37 °C in a water bath. At the end of the incubation, 400 μL of stop solution (200 ng/mL tolbutamide and 200 ng/mL labetalol in acetonitrile) was added to precipitate the protein, and the mixture was mixed thoroughly. Samples were then vortexed and centrifuged (20 min, 4000 rpm, 4 °C). After centrifugation, an Apricot automation workstation was used to transfer 50 μL of supernatant into 100 μL of HPLC water. The positive compounds, such as propantheline bromide (Lot#: R000190915), enalapril maleate salt (Lot#: MKBR8243V), bisacodyl (Lot#: 5-XJZ-44-1), procaine hydrochloride (Lot#: UCFUA-FH), were incubated in parallel with the BEA samples. Each bioanalysis plate was sealed and shaken for 10 min prior to LC-MS/MS analysis. The % remaining test compound after incubation in plasma was calculated using the following equation:

(11) $$\%\;\mathrm{Remaining}=\;\frac{\mathrm{PAR}\;\mathrm{at}\;\mathrm{appointed}\;\mathrm{incubation}\;\mathrm{time}}{\mathrm{PAR}\;\mathrm{at}\;T0\;\mathrm{time}}\times 100$$

where PAR is the peak area ratio of analyte versus internal standard (IS).

The appointed incubation time points are T0 (0 min), Tn (*n* = 0, 10, 30, 60, 120 min).

##### Metabolite Identification

The objectives of this study were to search for and to identify the metabolites of BEA in liver microsomes from mice, rats, dogs, monkeys and humans by LC-UV-MS*^n^* (*n* = 1~2) (based on peak intensity ≥ 1% of total drug related components), and to propose its metabolic pathways.

The test compound BEA at 10 µM was incubated with liver microsomes at 37 °C for 60 min. The positive control, 7-ethoxycoumarin (7-EC) at 10 µM, was run concurrently to assess Phase I metabolic activities in liver microsomes. The results indicate that the liver microsomes incubation system was reliable for metabolic study. After incubation, the samples were analyzed by LC-UV-MS (Table S7). The structures of the metabolites were proposed based on the interpretation of their MS and MS2 data. The results are presented in Table 6 and Figure 6.

$$\%Total=\frac{Peak\;Area\;of\;a\;Releted\;Component}{Peak\;Area\;of\;Total\;Releted\;Component}\times 100\%\;$$

#### 5.2.2. In Vivo Studies

##### PK Experiments in Rats

The PK of BEA were evaluated in cannulated male Sprague Dawley rats following an intravenous infusion (1 h) or oral dosing, with *N* = 3 for each route of administration. Sprague Dawley rats weighing 210 ± 10 g each were obtained from Vital River Laboratory Animal Technology Co., Ltd. (Beijing, China), and were used for pharmacokinetic assessment. All protocols involving animals were approved by the XBL-China Institutional Animal Care and Use Committee (IACUC No.2020-007). Animals were treated in accordance with the Animal Welfare Act and the “Guide for the Care and Use of Laboratory Animals” (NIH Publication 86-23, revised 1985). The animals were supplied with water and a commercial rodent diet ad libitum prior to study initiation. The rats were administered BEA as an intravenous infusion at a dose of 0.5 mg/kg and orally at a dose of 2 mg/kg, respectively. The animals were dosed via the appropriate route at time 0, and samples were collected at several time points up to 24 h following dose administration. Following intravenous infusion administration, blood samples were collected from the jugular vein cannula at predose as well as at 0.083, 0.25, 0.5, 1, 2, 4, 8, and 24 h postdose of BEA. Following oral dosing, blood samples were collected at predose as well as 0.25, 0.5, 1, 2, 4, 8, and 24 h postdose of BEA. The blood volume at each timepoint was 0.25 mL. Rat plasma samples were separated from the blood samples by centrifugation at 12,000 g for 15 min and stored in the freezer at −80 °C before analysis. Noncompartmental PK parameters such as area under the curve (AUC) and T_1/2_ were calculated using WinNonlin Professional (version 6.3, Pharsight, Sunnyvale, CA, USA). The absolute oral bioavailability of BEA in rats was calculated by the AUC(_0−_∞) ratio obtained following oral and i.v. administration.

(12) $$F=\frac{\mathrm{AUC}\left(0-\infty \right)p.o.}{\mathrm{AUC}\left(0-\infty \right)i.v.}\times \frac{Dose\;\left(i.v.\right)}{Dose\;\left(p.o.\right)}\times 100$$

##### PK Analysis

Blood concentration–time curves obtained for each compound were analyzed by means of noncompartmental PK analysis with WinNonlin Professional (version 6.3, Pharsight, Sunnyvale, CA, USA). Following intravenous infusion or bolus administration, the elimination T_1/2_, total AUC, AUC_0−t_, systemic blood clearance, and steady-state volume of distribution were calculated using the appropriate noncompartmental model (constant infusion, bolus intravenous injection, and extravascular, respectively). Following oral dosing, C_max_, T_max_, AUC_0−t_, and AUC_0−∞_ were calculated. The PK parameters were determined with WinNonlin software using the linear up/log down method in which the linear trapezoidal rule was used any time that the concentration increased, and the logarithmic trapezoidal rule was used any time that the concentration decreased. The summarized PK parameters are reported as the mean ± standard deviation.

## Figures and Tables

**Table 1:** Plasma protein binding of BEA in five species (*n* = 3) *^a^*.

Species	Human	Rat	Mouse	Dog	Monkey
Bound Fraction (%)	99.88 ± 3.53	99.93 ± 2.90	99.94 ± 0.57	99.92 ± 2.91	99.91 ± 1.62
Fu *^b^* (%)	0.12	0.07	0.06	0.08	0.09
Recovery (%)	96.64 ± 3.53	86.70 ± 2.90	87.97 ± 0.57	89.92 ± 2.91	82.54 ± 1.62

*^a^* Bound fraction values > 90% in plasma indicate high plasma protein binding; bound fraction values between 50 and 90% indicate moderate plasma protein binding; bound fraction values <50% indicate low plasma protein binding. *^b^* Fu (%), unbound fraction (%).

**Table 2:** CYP inhibition evaluation of BEA in human liver microsomes *^a^*(*n* = 3).

Test Compound Isozymes	IC_50_ (μM) *^b^*				
	CYP1A2	CYP2C9	CYP2C19	CYP2D6	CYP3A4
BEA	>50	>50	2.24	48.6	3.30
α-Naphthoflavone	0.011	ND	ND	ND	ND
Sulfaphenazole	ND	0.083	ND	ND	ND
(+)-*N*-3-benzylnirvanol	ND	ND	0.002	ND	ND
Quinidine	ND	ND	ND	0.045	ND
Ketoconazole	ND	ND	ND	ND	0.01

*^a^* IC50 < 10 μM—potent inhibitor; IC50 > 10 μM—weak inhibitor. *^b^* ND: not done.

**Table 3:** Half-life and intrinsic clearance of BEA in liver microsomes of five species (*n* = 2) *^a^*.

Species	HLM		RLM		MLM		DLM		CLM	
	Testosterone	BEA	Testosterone	BEA	Testosterone	BEA	Testosterone	BEA	Testosterone	BEA
T_1/2_ (min)	12.3	44.9	1.0	25.6	3.9	12.3	20.0	6.1	5.0	7.3
CL_int(mic)_ (μL/min/mg) *^a^*	112.8	30.9	1369.8	54.2	359.1	112.7	69.2	228.7	276.7	191.1
CL_int(liver)_ (mL/min/kg)	101.6	27.8	2465.7	97.6	1422.2	446.2	99.6	329.4	373.5	258.0
Remaining % (T = 60 min)	3.8	38.8	0.0	14.9	0.0	3.1	12.0	0.3	0.0	0.3

*^a^* Clint, intrinsic clearance; clearance rate >70% in whole hepatic blood flow indicates high clearance; clearance rate between 30 and 70% in whole hepatic blood flow indicates moderate clearance; clearance rate < 30% in whole hepatic blood flow indicates low clearance.

**Table 4:** Half-life and intrinsic clearance of BEA in hepatocytes of five species (*n* = 2).

Species	Human		Rat		Mouse		Dog		Monkey	
	7-Ethoxycoumarin	BEA	7-Ethoxycoumarin	BEA	7-Ethoxycoumarin	BEA	7-Ethoxycoumarin	BEA	7-Ethoxycoumarin	BEA
T1/2 (min)	41.6	83.3	18.2	75.6	<7.5	71.7	5.4	15.6	13.6	28.0
CLint (hep) (μL/min/10^6^) *^a^*	33.3	16.6	76.0	18.3	>184.8	19.3	256.4	89.0	102.0	49.4
CLint (liver) (mL/min/kg)	92.6	46.3	355.7	85.7	>2195.4	229.7	1763.9	612.5	367.4	178.0
Remaining % (T = 90 min)	21.9	40.9	2.1	39.8	0.0	41.7	0.0	1.7	0.0	10.5

*^a^* Clint, intrinsic clearance; clearance rate >70% in whole hepatic blood flow indicates high clearance; clearance rate between 30 and 70% in whole hepatic blood flow indicates moderate clearance; clearance rate <30% in whole hepatic blood flow indicates low clearance.

**Table 5:** Half-life and remaining of BEA in the plasma of five species (*n* = 2).

Species	Human	Rat	Mouse	Dog	Monkey
T_1/2_ (min)	>289	>289	>289	>289	>289
Remaining % (T = 120 min)	106.0	105.8	110.6	113.1	103.7

**Table 6:** Summary of BEA and its metabolites in mouse, rat, dog, monkey and human liver microsomes *^a^*.

Code	[M+H]^+^ *m*/*z*	RT *^b^* (min)	Formula	Mouse	Rat	Dog	Monkey	Human	Metabolic Pathways *^c^*
M1	1107.4960	7.61	C_55_H_74_N_6_O_16_S	+	+	+	+	-	Oxygenation, and glutathione conjugation (P + O + GSH)
M2	935.4095	7.91	C_48_H_62_N_4_O_13_S	+	+	+	+	+	Di-oxygenation, cysteine conjugation (P + 2O + Cys)
M3	832.4009	8.46	C_45_H_57_N_3_O_12_	+	+	+	+	-	Tri-oxygenation (P + 3O)
M4	832.4003	8.63	C_45_H_57_N_3_O_12_	+	+	+	+	+	Tri-oxygenation (P + 3O)
M5	832.4011	8.87	C_45_H_57_N_3_O_12_	+	+	+	+	+	Tri-oxygenation (P + 3O)
M6	802.3901	9.66	C_44_H_55_N_3_O_11_	+	+	+	+	+	Di-oxygenation, *N*-demethylation (P + 2O–CH_2_)
M7	816.4054	9.73	C_45_H_57_N_3_O_11_	+	+	+	+	+	Di-oxygenation (P + 2O)
M8	800.4105	9.95	C_45_H_57_N_3_O_10_	+	-	+	+	+	Mono-oxygenation (P + O)
M9	816.4052	10.28	C_45_H_57_N_3_O_11_	+	+	+	+	+	Di-oxygenation (P + 2O)
M10	800.4106	10.90	C_45_H_57_N_3_O_10_	+	+	+	+	+	Mono-oxygenation (P + O)
M11	816.4055	10.93	C_45_H_57_N_3_O_11_	+	+	+	+	+	Di-oxygenation (P + 2O)
M12	800.4113	10.97	C_45_H_57_N_3_O_10_	-	-	-	+	+	Mono-oxygenation (P + O)
M13	800.4108	11.30	C_45_H_57_N_3_O_10_	+	+	+	+	+	Mono-oxygenation (P + O)
M14	800.4105	11.99	C_45_H_57_N_3_O_10_	+	+	+	+	+	Mono-oxygenation (P + O)
M15	770.4002	12.75	C_44_H_55_N_3_O_9_	+	+	+	+	+	*N*-demethylation (P – CH_2_)
BEA	784.4156	13.01	C_45_H_57_N_3_O_9_	+	+	+	+	+	NA

*^a^* +: metabolite was detected in liver microsomes, -: metabolite was detected in liver microsomes. *^b^* RT: Retention time of LC-MS; *^c^* P: parent; GSH: C_10_H_15_N_3_O_6_S; Cys: C_3_H_5_NO_2_S; NA: not applicable.

**Table 7:** The pharmacokinetic parameters of BEA after oral or intravenous administration in rats (*n* = 3).

PK Parameters	Dosed (mg/kg)	C_max_ (ng/mL)	T_max_ (h)	AUC_0−t_ (ng·h/mL)	AUC_0−__∞_ (ng·h/mL)	CL (mL/min/kg)	*V*d (L/kg)	*T*_1/2_ (h)	F (%)
i.v.	0.5	454.0 ± 110.0	NA	339.0 ± 66.8	356.0 ± 66.7	23.9 ± 4.3	5.9 ± 2.5	5.1 ± 2.6	NA
p.o.	2	41.6 ± 0.6	1.0 ± 0	393.0 ± 54.6	421.0 ± 61.4	NA	NA	5.9 ± 0.6	29.5

## Data Availability

Not applicable.

## Supplementary Materials

The following are available online at https://www.mdpi.com/article/10.3390/toxins14070477/s1, Figure S1: Stability of positive compounds in plasma of five species. Table S1: The final concentration of each solution in CYP Inhibition studies. Table S2: Plasma protein binding of warfarin in five species (*n* = 3) a. Table S3: CYP Inhibition profiles of positive controls in Human Liver Microsomes (*n* = 3). Table S4: Half-life and remaining of BEA and positive compounds in plasma of five species (*n* = 2). Table S5: LC-MS method in PK Experiments. Table S6: LC-MS method in PPB Experiments, metabolic stability studies and CYP Inhibition Evaluation.
