# Supplementary material for: Study on In Vitro Metabolism and In Vivo Pharmacokinetics of Beauvericin
Source: Toxins (Basel). 2022 Jul 12;14(7):477. doi: 10.3390/toxins14070477 (PMC9320654; doi:10.3390/toxins14070477)
Supplement: Supplementary file 1 [file toxins-14-00477-s001.zip › toxins-1788002-supplementary.pdf]

Supplementary Materials: Study on In Vitro Metabolism and In Vivo Pharmacokinetics of Beauvericin

Yu Yuan, Guangpeng Meng, Yuanbo Li and Chunjie Wu

Table S1. The final concentration of each solution in CYP Inhibition studies.

**Preparati6ns of BEA working solution in CYP Inhibition test. (100× final conc.)**					
**Concentrations of test compounds**	**Volume of solution(μL)**	**1:1 DMSO: MeOH(μL)**	**Working Conc. (mM)**	**Final Conc. in assay (μM)**	
10 mM	30.0	30.0（MeOH）	5.00	50.0	
5 mM	30.0	70.0	1.50	15.0	
1.5 mM	30.0	60.0	0.500	5.000	
0.5 mM	30.0	70.0	0.150	1.500	
0.15 mM	30.0	60.0	0.0500	0.500	
0.05 mM	30.0	70.0	0.01500	0.1500	
0.015 mM	30.0	60.0	0.00500	0.0500	
**Preparations of positive controls solution in CYP Inhibition test (100× final conc.)**					
**Compounds**	**Stock Conc.(mM)**	**Vol. of solution (μL)**	**Vol. MeOH (μL)**	**Working Conc.(μM)**	**Final Conc.(μM)**
α-Naphthoflavone	3	10	90	300	3
Sulfaphenazole	3	10	90	300	3
(+)-N-3-benzylnirvanol	1	10	90	100	1
Quinidine	3	10	90	300	3
Ketoconazole	3	10	90	300	3
**Preparations of cocktail substrate solution in CYP Inhibition test. (10× final conc.)**					
**CYP**	**Substrate**	**Stock Conc. (mM)**	**Working Conc. (μM)**	**Final Conc. (μM)**	**Vol.(μL)**
1A2	Phenacetin	20	100	10	50
2C9	Diclofenac	10	50	5	50
2C19	S-mephenytoin	20	300	30	150
2D6	Dextromethorphan	20	50	5	25
3A4	Midazolam	10	20	2	20
	**PB**	100 mM			9705
				**Total vol:**	**10.0 mL**

Table S2. Plasma protein binding of warfarin in five species (n=3) [a].

**Species**	**Human**	**Rat**	**Mouse**	**Dog**	**Monkey**
Fraction of Bound (%)	98.86±4.99	99.48±5.60	97.29±6.32	97.54±7.2	99.16±9.69
Fu[b] (%)	1.14	0.52	2.71	2.46	0.84
Recovery (%)	108.22±4.99	102.44±5.6	100.67±6.32	106.80±7.24	100.97±9.69

[a] Bound fraction values > 90% in plasma indicated high plasma protein binding; bound fraction values between 50-90% indicated moderate plasma protein binding; bound fraction values < 50% indicated low plasma protein binding.

Table S3. CYP Inhibition profiles of positive controls in Human Liver Microsomes（n=3）.

**Compounds**	**IC_{50} (μM)**	**Activity (% VC)**	**Inhibition(%)**
α-Naphthoflavone	0.011	12.8	87.2
Sulfaphenazole	0.083	20.0	80.0
(+)-*N*-3-benzylnirvanol	0.002	16.2	83.8
Quinidine	0.045	4.8	95.2
Ketoconazole	0.01	1.52	98.5

Table S4. Half-life and remaining of BEA and positive compounds in plasma of five species (n=2).

**Test compound**	**$T_{1/2}$ (min)**					**Remaining % (T=120min)**				
	**human**	**rat**	**mouse**	**dog**	**monkey**	**human**	**rat**	**mouse**	**dog**	**monkey**
**BEA**	>289	>289	>289	>289	>289	106.0	105.8	110.6	113.1	103.7
**Propantheline bromide**	**10.3**	**ND**	**36.6**	**ND**	**ND**	0.0	ND	9.7	ND	ND
**Enalapril maleate salt**	**ND**	**12.8**	**ND**	**ND**	**ND**	ND	0.2	ND	ND	ND
**Bisacodyl**	**ND**	**ND**	**ND**	**7.2**	**ND**	ND	ND	ND	0.0	ND
**Procaine Hydrochloride**	**ND**	**ND**	**ND**	**ND**	**4.4**	ND	ND	ND	ND	0.0

[a] ND: not done.

Table S5. LC-MS method in PK Experiments.

**Instrument**	6500_Triple Quad 6500 plus	
**Matrix**	Male SD Rat Plasma(EDTA-K2)	
**Analyte(s)**	BEA	
**Internal standard(s)**	IS1:5 in 1 internal standard in ACN, 100 ng/mL for each)	
**MS conditions**	ESI: Positive	
	SRM detection	
	BEA:[M+H]+m/z: 784.50/244.40 Da	
	Glyburide:[M+H]+m/z: 494.20/169.10 Da	
**UPLC conditions**	Mobile Phase:	
	Mobile Phase A:0.1% FA & 2mM NH4OAc in water/ACN (v:v, 95:5)	
	Mobile Phase B:0.1% FA & 2mM NH4OAc in ACN/water (v:v, 95:5)	
	Time (min)	Mobile Phase B (%)
	Initial	25
	0.2	25
	0.7	45
	1.2	98

1.6	98
1.61	25
1.8	25
Column:ACQUITY UPLC Protein BEH C4 300Å 1.7 μm 2.1 × 50 mm Column	
Column temperature:45 C	
Flow rate:0.6 mL/min	
Retention time:	
BEA:1.37 min	
Glyburide:1.11 min	

Table S6. LC-MS method in PPB Experiments, metabolic stability studies and CYP Inhibition Evaluation.

**Instrument**	6500_Triple Quad 6500 plus	
**Matrix**	PPB plasma samples, Metabolic Stability samples, CYP Inhibition Evaluation samples	
**Analyte(s)**	BEA	
**MS conditions**	ESI: Positive	
	SRM detection	
	BEA:[M+H]+m/z: 784.50/244.40 Da	
**UPLC conditions**	Mobile Phase:	
	Mobile Phase A:0.1% FA in water/ACN (v:v, 95:5)	
	Mobile Phase B:0.1% FA in ACN/water (v:v, 95:5)	
	Time (min)	Mobile Phase B (%)
	Initial	35
	0.01	95
	0.3	95
	1.3	35
	1.31	35
	2	35
	Column: ACQUITY UPLC HSS T3 1.8μm(50 mm×2.10 mm)	
	Column temperature:45 C	
	Flow rate:0.6 mL/min	
	Retention time:	
	BEA:1.14 min	

Table S7. LC-MS method in Metabolite Identification.

LC-UV-MS condition for positive control

LC System:	Waters Acquity UPLC
Column:	Waters Acquity UPLC HSS T3, 2.1×100 mm, 1.8 μm
UV Detector:	λ: 190~400 nm

Column Temperature:	40 ℃
Autosampler Temperature:	8 ℃
Mobile Phase :	0.1% FA and 2 mM ammonium formate in H_2O/ACN (v:v = 95:5)
	0.1% FA and 2 mM ammonium formate in H_2O/ACN (v:v = 5:95)
Flow Rate:	0.5 mL/min
Injection Volume:	5 μL

Gradient:

Step	Time (min)	A (%)	B (%)
0	0.01	98.0	2.0
1	0.50	98.0	2.0
2	4.00	85.0	15.0
3	5.50	75.0	25.0
4	6.50	75.0	25.0
5	7.50	10.0	90.0
6	8.50	10.0	90.0
7	8.51	98.0	2.0
8	9.50	98.0	2.0

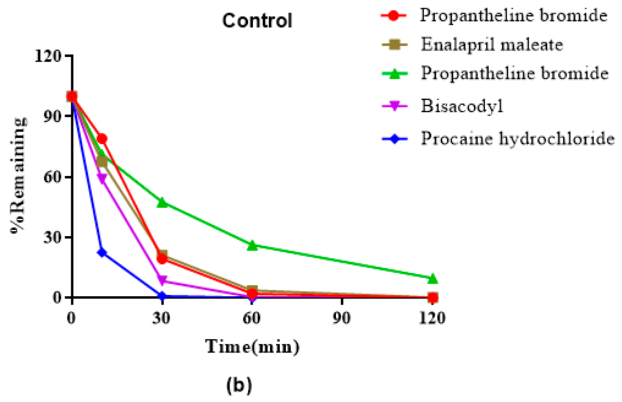

Figure S1. Stability of positive compounds in plasma of five species.
